# Patients readmitted to intensive care: who they are and what happens to them?

**DOI:** 10.1186/cc10206

**Published:** 2011-06-22

**Authors:** AP Nassar, LD Salles, L Brauer

**Affiliations:** 1 Hospital e Maternidade São Camilo, Unidade Pompeia, São Paulo - SP, Brazil

## Introduction

There is growing interest in quality-of-care indicators in the ICU. Readmission is one of the proposed indicators to be measured.

## Objective

To investigate the incidence of, outcomes and possible risk factors for readmission in a large cohort of patients in a medical-surgical ICU and to evaluate the accuracy of Simplified Acute Physiology Score III (SAPS III) and Acute Physiologic and Chronic Health Evaluation IV (APACHE IV) to predict readmissions.

## Methods

We conducted an analysis of prospectively collected data from all patients admitted between January 2009 and December 2010 who survived their first ICU stay. Patients aged <18 years, patients transferred to another hospital and those who were not yet discharged until 1 February 2011 were excluded from the analysis. The following variables were evaluated as possible risk factors for readmission: sex, age, type of admission (medical vs. surgical), SAPS III, APACHE III score, APACHE IV mortality predicted risk, ICU length of stay (LOS), ICU discharge at night and on weekends. Accuracies of SAPS III and APACHE IV mortality predicted risk were assessed by calculating the area under the receiver operating characteristic curve. Categorical variables are presented as absolute numbers and percentages. Continuous variables are presented as medians and interquartile ranges.

## Results

A total of 3,993 patients were admitted during the study period and 3,637 fulfilled study inclusion criteria. Two hundred and eighty-three (7.8%) had at least one readmission. Patients' characteristics are displayed in Table 1. In the multivariate analysis, SAPS III (OR = 1.020; *P *= 0.008), APACHE III score (OR = 1.015; *P *< 0.001).

**Table 1:** Characteristics and outcomes of readmitted and nonreadmitted patients to ICU

	Readmitted patients (*n *= 283)	Nonreadmitted patients (*n *= 3,354)	*P *value
Male sex	142 (50.2)	1,722 (51.3)	0.707
Age	73 (56 to 82)	63 (47 to 77)	<0.001
Type of admission			0.379
Medical	243 (85.9)	2,813 (83.9)	
Surgical	40 (14.1)	541 (16.1)	
SAPS III	48 (38 to 57)	41 (33 to 49)	<0.001
APACHE III score	36.5 (23 to 51)	26 (18 to 36)	<0.001
APACHE IV risk (%)	8.36 (3.07 to 18.51)	2.94 (1.18 to 7.10)	<0.001
ICU LOS	3.92 (2.20 to 8.39)	2.45 (1.58 to 3.85)	<0.001
Discharge at night	62 (21.9)	589 (17.6)	0.068
Discharge on weekend	67 (23.7)	939 (28.0)	0.119
In-hospital mortality	81 (28.6)	43 (1.3)	<0.001

## Conclusion

Readmitted patients were older, had longer ICU LOS and higher severity scores at admission. Readmission was an independent factor associated with in-hospital mortality. SAPS III and APACHE IV at first admission had only moderate ability to predict readmissions.

